# Cholesterol consumption and risk of endometrial cancer: a systematic review and dose-response meta-analysis of observational studies

**DOI:** 10.18632/oncotarget.7913

**Published:** 2016-03-04

**Authors:** Ting-Ting Gong, Da Li, Qi-Jun Wu, Ya-Zhu Wang

**Affiliations:** ^1^ Department of Obstetrics and Gynecology, Shengjing Hospital of China Medical University, Shenyang, China; ^2^ Department of Clinical Epidemiology, Shengjing Hospital of China Medical University, Shenyang, China; ^3^ Department of Hematology, The First Affiliated Hospital of China Medical University, Shenyang, China

**Keywords:** cholesterol, endometrial cancer, epidemiology, meta-analysis

## Abstract

*In vivo* and *in vitro* studies have indicated the link of cholesterol consumption and endometrial cancer risk, however, previous observational studies have yielded inconsistent results. Additionally, a previous meta-analysis published in 2007 found limited evidence of aforementioned association. Therefore, we performed the dose-response meta-analysis to address this concern. Studies were identified using the PubMed, EMBASE and Web of Science databases from the database inception to the end of June 2015 as well as by examining the references of retrieved articles. Two authors independently performed the eligibility evaluation and data extraction. The summary risk estimates and 95% confidence intervals (CIs) were summarized by the random-effects models. One cohort and nine case-control studies were included in the dose-response analyses. Risk of endometrial cancer increased by 6% for 100 mg/day increment in the dietary consumption of cholesterol (Odds ratio (OR) = 1.06; 95% CI = 1.00–1.12), with significant heterogeneity (*I*^2^ = 64.2, *P* = 0.003). When stratified by study design, the result was significant in case-control studies (OR = 1.07; 95% CI = 1.01–1.13). Additionally, although the direction of the associations were consistent in the subgroup analyses stratified by study characteristics and adjustment for potential confounders, not all of them showed statistical significance. In summary, findings of the present dose-response meta-analysis partly support the positive association between dietary cholesterol consumption and risk of endometrial cancer. Since only one cohort study was included, more prospective studies and pooled analysis of observational studies are warranted to confirm our findings in the future.

## INTRODUCTION

Endometrial cancer (EC) is the fifth most common cancer among women worldwide with almost 0.32 million new cases diagnosed in 2012 which accounted for 4.8% of all female cancer cases [1]. Besides these well-established risk factors including obesity, change of endogenous hormones, and use of exogenous hormones [2–5], diet may also mediate endogenous estrogen levels, promoting endometrial carcinogenesis [6]. The continuous update project of World Cancer Research Fund and American Institute for Cancer Research (WCRF/AICR) concluded that there was limited evidence suggesting that non-starchy vegetables protect against EC, and that red meat was the cause of this cancer [7].

Experimental studies have proposed that several components of diet, especially lipids including saturated fatty acid, unsaturated fatty acid, and cholesterol intake have been proposed to influence EC risk by modulating the production, metabolism, and excretion of endogenous hormones [8–12]. We recently published a dose-response meta-analysis but found limited evidence of the association between dietary saturated, monounsaturated, and polyunsaturated fatty acids consumption and EC risk [13]. The summary relative risk for an intake increment of 10g/day was 1.02 (95% CI = 0.97–1.08; *I*^2^ = 66.0%) for saturated fatty acids, 0.98 (95% CI = 0.96–1.001; *I*^2^ = 0%) for monounsaturated fatty acids, and 1.00 (95% CI = 0.95–1.06; *I*^2^ = 0%) for polyunsaturated fatty acids intake [13]. Whether there is a association between dietary cholesterol intake and EC risk has still remained unknown. Previous observational studies yielded inconsistent results of the dietary cholesterol consumption and EC risk. Some published studies indicated statistically significant increased risks [14–16], but other studies cast doubt on the strength of the positive aforementioned association [17–23]. Results of the most-recent meta-analysis which was published in 2007 including 6 case-control studies showed an odds ratio (OR) of 1.39 (95% confidence interval (CI) = 0.97–2.00, *I*^2^ = 62.3%, *P* for heterogeneity = 0.02) and 1.35 (95% confidence interval (CI) = 0.96–1.90, *I*^2^ = 68.3%, *P* for heterogeneity < 0.01) for the highest compared with the lowest intakes and dose-response analysis of per 150 mg/1000 kcal dietary cholesterol consumption, respectively [24]. However, since limited included studies of this meta-analysis, the authors failed to carry out subgroup analyses to find the source of heterogeneity. Additionally, it has not been clear whether the findings of the study were robust in the subgroup and sensitivity analyses. Notably, the evidence of cholesterol intake and EC risk was absent not only in the report of WCRF/AICR in 2007 [25] but also in the continuous update project of WCRF/AICR including studies up to December 2012 [7]. Several observational studies including one of the largest population-based cohort studies, the European Prospective Investigation into Cancer and Nutrition (EPIC), have been published on cholesterol intake in relation to EC risk since the previous meta-analysis [14, 15, 17, 18]. Therefore, in an attempt to update this evidence, we carried out an updated dose-response meta-analysis of the published studies.

## RESULTS

### Identification of studies

**Figure 1:**
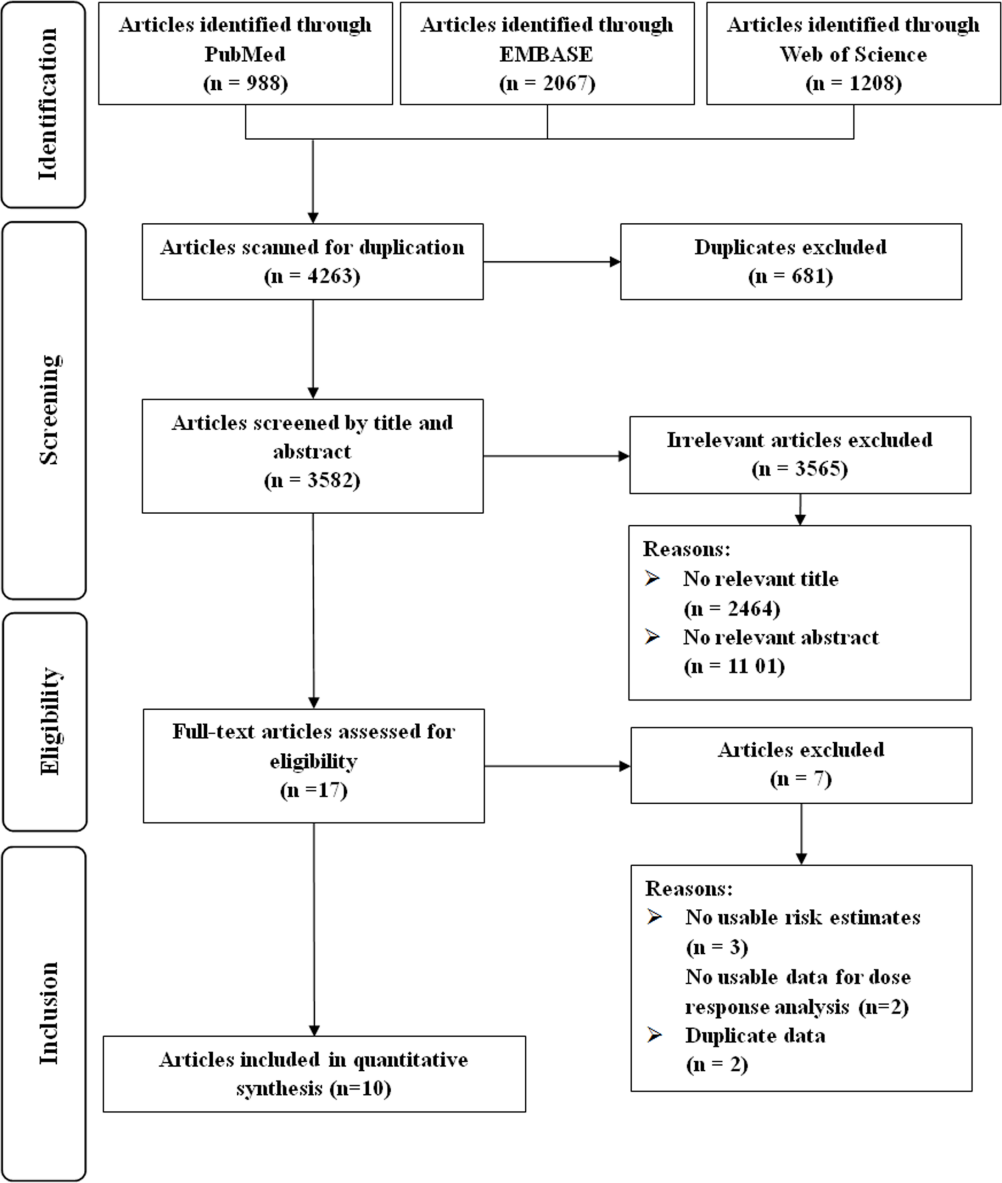
Flow-chart of study selection

Our initial search yielded a total of 4263 unique articles (Figure 1). After two rounds of reviews and searching citations of retained articles, we identified 17 articles for full-text review. We excluded articles: i) without risk estimates or 95% CIs and ii) with duplicated study populations. Thus ten observational studies were eligible for inclusion [14–23].

### Study characteristics and quality assessment

The characteristics of these included studies are summarized in Table 1. The studies were published between 1993 and 2015 and included a total of 4512 EC cases and 305,374 non-cases from individual studies. One of these ten studies was cohort study [17] and the other nine were case-control studies [14–16, 18–23]. Three of these studies were conducted in Europe [15, 17, 22] and seven in North America [14, 16, 18–21, 23]. Of nine case-control studies, control subjects were drawn from the general populations in four studies [14, 16, 20, 21] and hospitals in five studies [15, 18, 19, 22, 23]. All studies used self-administered food frequency questionnaires (FFQ) to assess diet, and the FFQ was validated in five studies [14, 15, 17, 19, 21].

**Table 1:** Characteristics of studies included in the meta-analysis

First author (ref), year, Country	No. of cases/cohort (controls), age, follow-up	Cholesterol categories(Dietary assessment)	Risk estimates (95% CI)	Matched/adjusted factors
**Prospective study**				
Merritt et al [17], 2015, Europe	1303/301,107 (25–70y), 11y	Quartile 1 Quartile 2 Quartile 3 Quartile 4 (Validated FFQ)	1.00 (Ref) 0.97 (0.82–1.16) 1.00 (0.83–1.20) 1.00 (0.83–1.20) **Hazard ratio**	BMI, total energy intake, smoking status, age at menarche, OC use, parity, and a combined variable for menopausal status and postmenopausal hormone use and were stratified by age and study center
**Case-control study**				
Biel et al [14], 2011, Canada, PC-CS	506/981 (mean, 58.7/58.3y)	Quartile 1 Quartile 2 Quartile 3 Quartile 4 (Validated FFQ)	1.00 (Ref) 1.22 (0.87–1.72) 1.29 (0.92–1.82) 1.51 (1.08–2.11) **Odds Ratio**	Age, total energy intake, age at menarche, BMI, parity, educational level, hypertension history, OC use, hormone therapy use combined with menopausal status, and alcohol consumption
Yeh et al [18], 2009, USA, HC-CS	541/541 (mean, 63.3/63.2y)	Quartile 1 Quartile 2 Quartile 3 Quartile 4 (FFQ)	1.00 (Ref) 0.90 (0.61–1.31) 1.34 (0.89–2.00) 1.09 (0.65–1.85) **Odds Ratio**	Age, BMI, exogenous estrogen use, smoking, total menstrual months, total energy, total protein and carbohydrates intake
Lucenteforte et al [15], 2008, Italy, HC-CS	454/908 (median, 60/61y)	Quintile 1 Quintile 2 Quintile 3 Quintile 4 Quintile 5 (Validated FFQ)	1.00 (Ref) 1.10 (0.70–1.70) 1.20 (0.80–1.80) 1.60 (1.00–2.40) 2.10 (1.40–3.20) **Odds Ratio**	Age, study centre, year of interview, education, PA, BMI, history of diabetes, age at menarche, age at menopause, parity, OC use, hormone replacement therapy use, total energy intake, according to the residual models
Salazar-Martinez et al [19], 2005, Mexico, HC-CS	85/629 (mean, 51.7/57.1y)	Tertile 1 Tertile 2 Tertile 3 (Validated FFQ)	1.00 (Ref) 0.76 (0.41–1.45) 0.81 (0.37–1.76) **Odds Ratio**	Age, total energy intake, number of live births, BMI, PA, and diabetes
Littman et al [20], 2001, USA, PC-CS	679/944 (45–74y)	Quintile 1 Quintile 2 Quintile 3 Quintile 4 Quintile 5 (FFQ)	1.00 (Ref) 1.10 (0.75–1.50) 1.40 (0.94–2.00) 1.30 (0.86–1.90) 1.30 (0.85–2.10) **Odds Ratio**	Age, county of residence, total energy intake, unopposed estrogen use, cigarette smoking, and BMI
McCann et al [21], 2000, USA, PC-CS	232/639 (mean, 63.5/55.9y)	Quartile 1 Quartile 2 Quartile 3 Quartile 4 (Validated FFQ)	1.00 (Ref) 0.80 (0.50–1.20) 0.50 (0.30–0.90) 0.70 (0.40–1.40) **Odds Ratio**	Age, education, BMI, diabetes, hypertension, pack-years cigarette smoking, age at menarche, parity, OC use, menopause status, postmenopausal estrogen use, and total energy intake
Tzonou et al [22],* 1996, Greece, HC-CS	145/298 (N/A)	Quartile 1 Quartile 2 Quartile 3 Quartile 4 (FFQ)	1.00 (Ref) 1.03 (0.58–1.82) 1.03 (0.58–1.82) 1.31 (0.75–2.29) **Odds Ratio**	Age
Potischman et al [16], 1993, USA, PC-CS	399/296 (mean, 59.1/58y)	Quartile 1 Quartile 2 Quartile 3 Quartile 4 (FFQ)	1.00 (Ref) 1.50 (0.90–2.40) 0.80 (0.50–1.50) 2.00 (1.20–3.30) **Odds Ratio**	Age, BMI, current smoking, years of education, number of births, ever OC use, ever menopausal estrogen use, and total calories intake
Barbone et al [23], 1993, USA, HC-CS	168/334 (mean, 64/63y)	Tertile 1 Tertile 2 Tertile 3 (FFQ)	1.00 (Ref) 1.10 (0.60–2.20) 1.60 (0.80–2.90) **Odds Ratio**	Age, race, years of schooling, total calories, use of unopposed estrogens, obesity, shape of obesity, smoking, age at menarche, age at menopause, number of pregnancies, diabetes, and hypertension

BMI, body mass index; CI, confidence interval; HC-CS, hospital-based case-control study; PA, physical activity; PC-CS, population-based case-control study; N/A, not available; OC, oral contraceptive; FFQ, food frequency questionnaire.

* Risk estimates were calculated from published data with EpiCalc 2000 software (version 1.02; Brixton Health).

Table 2 and Table 3 presented the quality of these included studies on the basis of the Newcastle-Ottawa Quality Assessment Scale (NOS). Briefly, the only one cohort study was assigned all scores of the assessment. Among case-control studies, four studies [14, 16, 20, 21] were assigned a star in the selection of control subjects category because the controls included in the studies came from the same population as the cases. One case-control study [22] was not assigned 2 stars in the control for important factors or additional factors category because they did not adjust for more than 2 important confounders in the multivariable analysis. For the exposure assessment category, four case-control studies [14, 15, 19, 21] were assigned a star because their food frequency questionnaires were validated. For the non-response rate category, five case-control studies [14, 16, 18, 19, 23] were not assigned a star because there were differences in response rates between cases and controls.

**Table 2:** Methodological quality of prospective studies included in the dose-response meta-analysis*

First author (reference), publication year	Representa-tivenessof the exposed cohort	Selection of the unexposed cohort	Ascertainment of exposure	Outcome of interest not present at start of study	Control for important factor or additional factor ^†^	Assessment of outcome	Follow-up long enough for outcomes to occur^‡^	Adequacy of follow-up of cohorts ^§^
Merritt et al [17], 2015	*	*	*	*	**	*	*	*

* A study could be awarded a maximum of one star for each item except for the item Control for important factor or additional factor. The definition/explanation of each column of the Newcastle-Ottawa Scale is available from (http://www.ohri.ca/programs/clinical_epidemiology/oxford.asp).

† A maximum of 2 stars could be awarded for this item. Studies that controlled for total energy intake received one star, whereas studies that controlled for other important confounders such as body mass index, reproductive factors received an additional star.

‡ A cohort study with a follow-up time > 10 y was assigned one star.

§ A cohort study with a follow-up rate > 75% was assigned one star.

**Table 3:** Methodological quality of case-control studies included in the dose-response meta-analysis*

First author (reference), publication year	Adequate definition of cases	Representa-tiveness of cases	Selection of control subjects	Definition of control subjects	Control for important factor or additional factor^†^	Exposure assessment	Same method of ascertainment for all subjects	Non response Rate^‡^
Biel et al [14], 2011	*	*	*	*	**	*	*	—
Yeh et al [18], 2009	*	*	—	*	**	—	*	—
Lucenteforte et al [15], 2008	*	*	—	*	**	*	*	*
Salazar-Martinez et al [19], 2005	*	*	—	*	**	*	*	—
Littman et al [20], 2001	*	*	*	*	**	—	*	*
McCann et al [21], 2000	*	*	*	*	**	*	*	*
Tzonou et al [22], 1996	*	*	—	*	—	—	*	*
Potischman et al [16], 1993	*	*	*	*	**	—	*	—
Barbone et al [23], 1993	*	*	—	*	**	—	*	—

* A study could be awarded a maximum of one star for each item except for the item Control for important factor or additional factor. The definition/explanation of each column of the Newcastle-Ottawa Scale is available from (http://www.ohri.ca/programs/clinical_epidemiology/oxford.asp).

† A maximum of 2 stars could be awarded for this item. Studies that controlled for total energy intake received one star, whereas studies that controlled for other important confounders such as body mass index, reproductive factors received an additional star.

‡ One star was assigned if there was no significant difference in the response rate between control subjects and cases by using the chi-square test (*P* > 0.05).

### Dose-response analysis of cholesterol consumption

**Figure 2:**
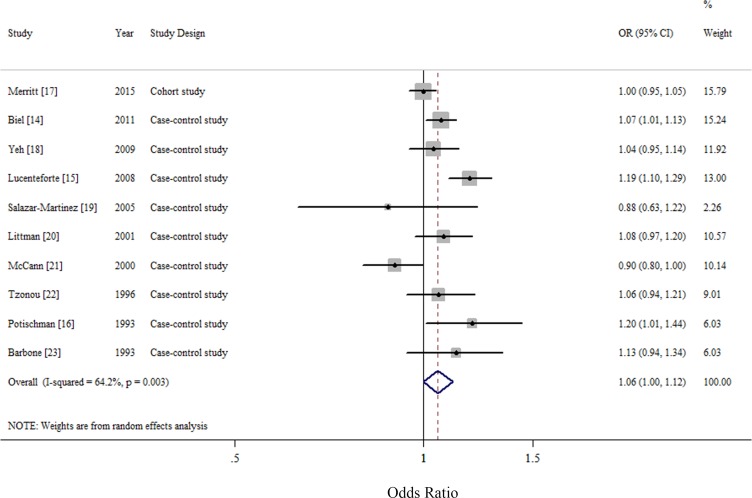
Forest plots (random effect model) of cholesterol consumption (per 100 mg/day) and endometrial cancer risk Squares indicate study-specific risk estimates (size of the square reflects the study-specific statistical weight); horizontal lines indicate 95% confidence interval; diamond indicates the summary relative risk with its 95% confidence interval. OR: odds ratio.

**Figure 3:**
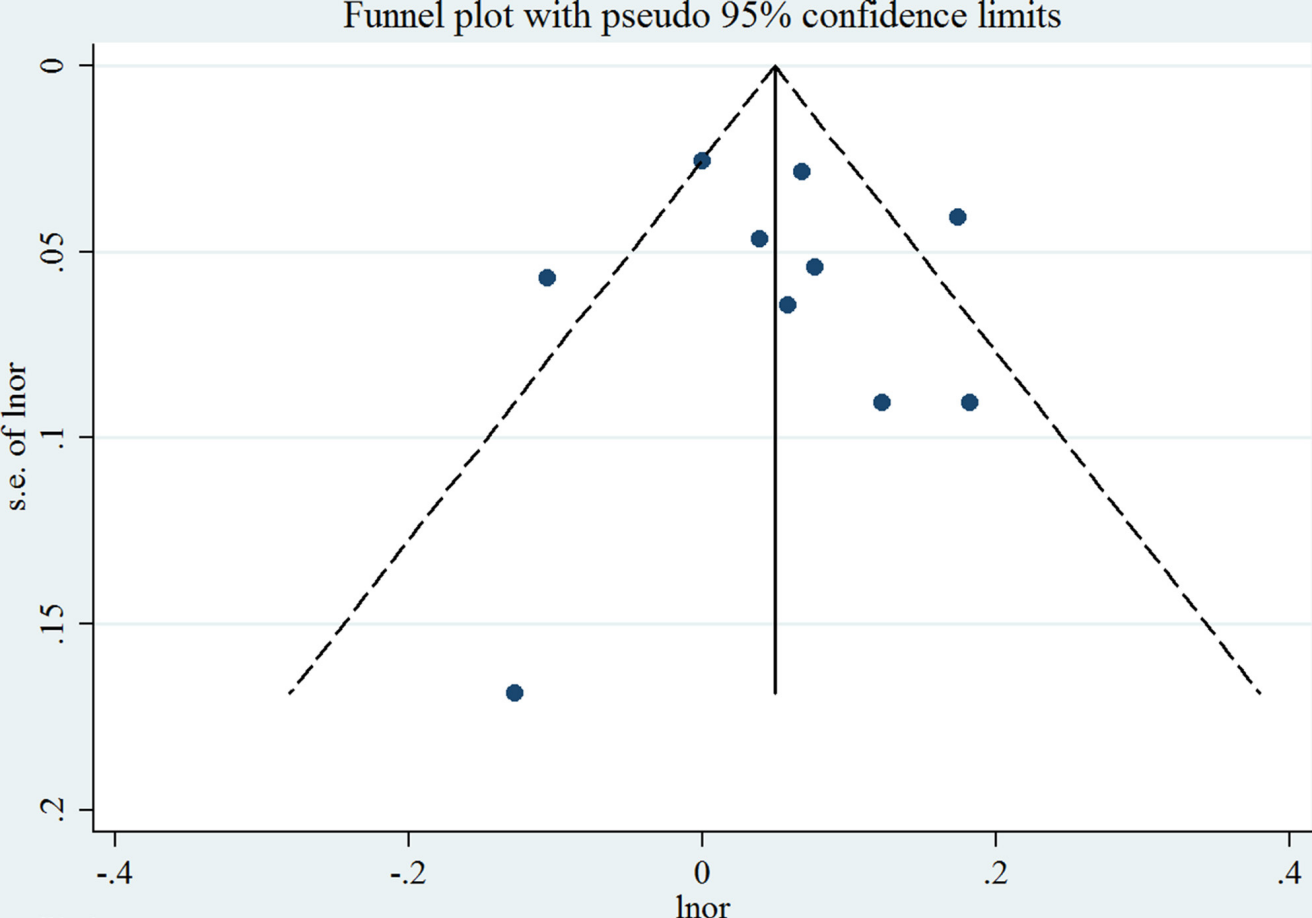
Funnel plot corresponding to the random-effects meta-analysis of the relationship between cholesterol consumption (per 100 mg/day) and endometrial cancer risk Lnor: ln odds ratio. SE: standard error.

For an increase intake of 100 mg/day, the summary OR of EC was 1.06 (95% CI = 1.00–1.12), with significant heterogeneity (*I*^2^ = 64.2%, *P* for heterogeneity = 0.003) (Figure 2). We flexibly modeled the dose-response relationships using restricted cubic splines and found no significant departure from linearity (*P* for nonlinearity = 0.67) (Supplementary Figure S1). The Egger's (*P* for bias = 0.785) and Begg's test (*P* for bias = 0.858) showed no evidence of publication bias as well as visual inspection of the funnel plot (Figure 3).

### Subgroup and sensitivity analysis

Table 4 presented the summary ORs and 95% CIs of associations between cholesterol consumption and EC risk in strata of selected factors. The direction of these associations were consistent in the subgroup analyses but not all of them showed statistical significance. Notably, when stratified by study design, since one cohort study was included, we found significant result in case-control studies (summary OR = 1.07; 95% CI = 1.01–1.13), with significant heterogeneity (*I*^2^ = 59.5%, *P* for heterogeneity = 0.011). Additionally, the results of meta-regression analyses did not show statistical significance.

**Table 4:** Summary risk estimates of the association between dietary cholesterol intake and endometrial cancer risk, dose-response analysis (per 100 mg/day increment)

	No. of study	Summary OR (95% CI)	*I*^2^ value (%)	*P*_h_*	*P*_h_**
**Overall**	10	1.06 (1.00–1.12)	64.2	< 0.01	
**Study design**					0.43
Cohort study	1	1.00 (0.95–1.05)	N/A	N/A	
Case-control study	9	1.07 (1.01–1.13)	59.5	0.01	
**Type of control subjects**					0.55
Population-based	4	1.05 (0.95–1.15)	70.6	0.02	
Hospital-based	5	1.09 (1.01–1.18)	45.4	0.12	
**Geographic location**					0.64
North America	7	1.05 (0.98–1.11)	50.1	0.06	
Europe	3	1.08 (0.96–1.22)	84.8	< 0.01	
**Validated FFQ**					0.31
Yes	6	1.03 (0.96–1.11)	77.0	< 0.01	
No	4	1.10 (1.03–1.18)	0	0.69	
**Number of cases**					0.55
≥ 450	5	1.07 (1.01–1.14)	70.8	< 0.01	
< 450	5	1.04 (0.92–1.17)	61.8	0.03	
**Adjustment for potential confounders**					
**Total energy intake**					0.98
Yes	9	1.06 (1.00–1.12)	68.2	< 0.01	
No	1	1.06 (0.94–1.21)	N/A	N/A	
**Body mass index**					0.69
Yes	8	1.05 (0.99–1.12)	71.4	< 0.01	
No	2	1.08 (0.98–1.20)	0	0.57	
**Cigarette smoking**					0.33
Yes	7	1.04 (0.99–1.10)	55.3	0.04	
No	3	1.10 (0.97–1.25)	57.9	0.09	
**Parity**					0.84
Yes	8	1.05 (0.99–1.13)	71.8	< 0.01	
No	2	1.07 (0.99–1.16)	0	0.82	
**Oral contraceptive use**					0.62
Yes	7	1.07 (1.00–1.14)	75.0	< 0.01	
No	3	1.04 (0.97–1.12)	0	0.59	
**Menopausal status**					0.86
Yes	7	1.06 (0.99–1.13)	74.8	< 0.01	
No	3	1.06 (0.98–1.15)	0	0.51	
**Hormone replacement therapy use**					0.65
Yes	8	1.06 (1.00–1.13)	70.9	< 0.01	
No	2	1.03 (0.91–1.17)	5.9	0.30	

CI, confidence interval; FFQ, food frequency questionnaire; N/A, not available; OR, odds ratio.

* *P*-value for heterogeneity within each subgroup.

** *P*-value for heterogeneity between subgroups with meta-regression analysis.

**Figure 4:**
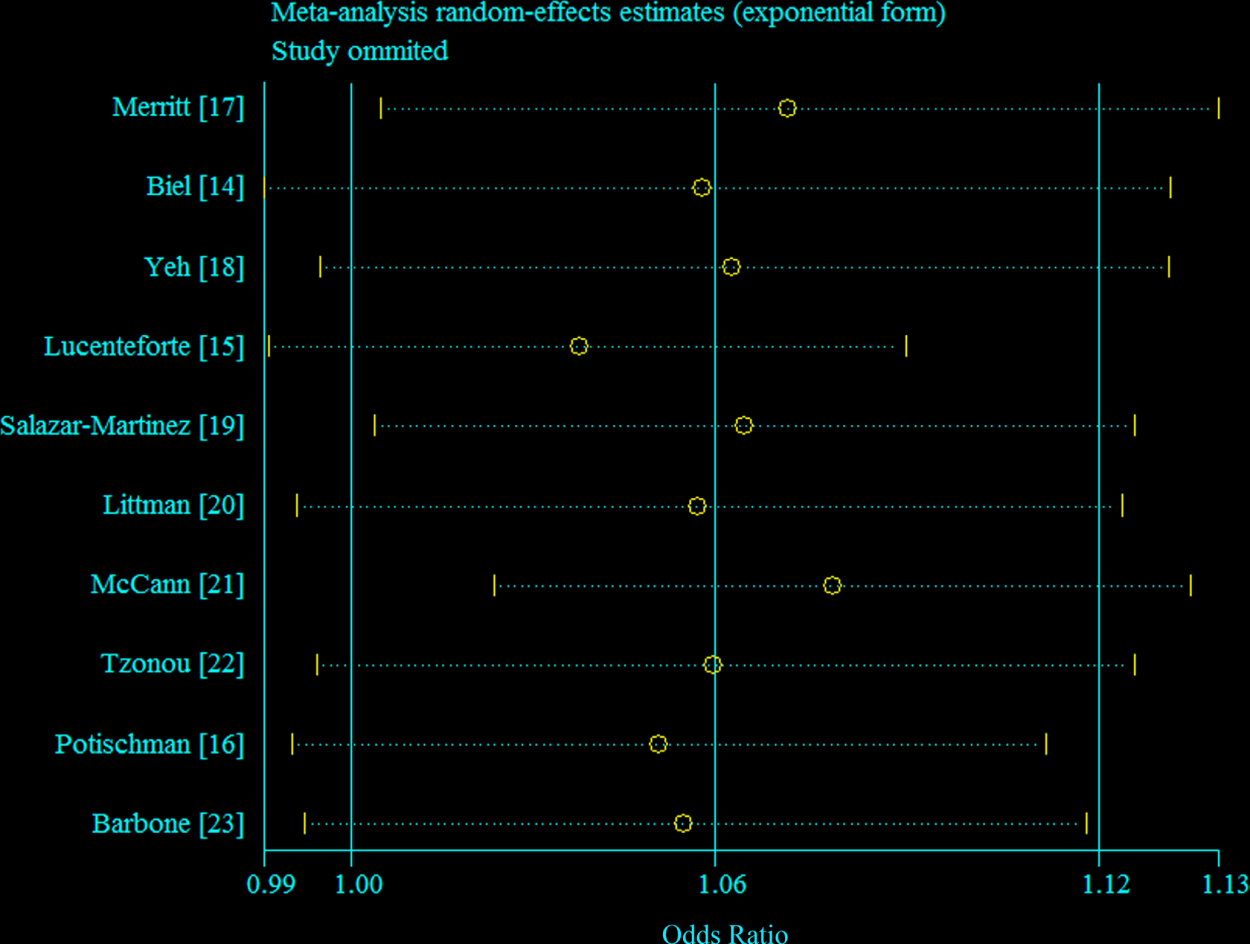
Galbraith plot corresponding to the relationship between cholesterol consumption and endometrial cancer risk Circles indicate the summary relative risk; dash line indicate 95% confidence interval.

In sensitivity analyses around the assignment of the dose of the top categories of consumption in studies that did not report median values, the summary OR was not changed (OR = 1.02 for every 100 mg per day increment in consumption of cholesterol). Figure 4 demonstrated the 10 study-specific RRs ranged from a low of 1.04 (95% CI = 1.00–1.09, *I*^2^ = 44.7%, *P* for heterogeneity = 0.07) after omitting the study by Lucenteforte et al. [15] to a high of 1.08 (95% CI = 1.02–1.13, *I*^2^ = 53.7%, *P* for heterogeneity = 0.03) after omitting the study by McCann et al. [21].

## DISCUSSION

In the systematic review and meta-analysis, we systematically reviewed and summarized the relationships between cholesterol consumption and incidence of EC. Our linear dose-response meta-analyses of 10 observational studies found that an increase in cholesterol consumption by 100 mg/day was associated with an 6% increased risk of EC. When stratified by study design, significant results were only observed among case-control studies. In addition, although the direction of this association was consistent in the other subgroup analyses, not all of them showed statistical significance.

EC benefits of low cholesterol consumption have been supported by several lines of evidence. Cholesterol and estrogen are physiologically interconnected [14]. As the major substrate leading to steroid hormone synthesis [26], cholesterol, can be converted to estrogen through a variety of metabolic pathways. Hence, increased concentration of cholesterol may influence EC risk by increasing bio-available estrogen synthesis [14]. Notwithstanding most cholesterol is produced by the liver, prolonged and high consumption of cholesterol raises the average serum cholesterol level [27]. Furthermore, increased dietary intake of cholesterol may be associated with increased oxidant stress reflected in higher levels of cholesterol oxidation products [28]. A previous pilot study demonstrated that plasma levels of cholesterol β-epoxide were significantly higher among EC cases than controls [29].

Previous experimental studies suggested that circulating cholesterol levels were decreased as a result of estrogen therapy in postmenopausal women [30]. In contrast, estrogen production occurs in the ovaries and circulating estrogen levels are tightly regulated during pre-menopause [31]. Therefore, the effect of cholesterol on estrogen levels may be marginal [31]. The relative influence of cholesterol on estrogen bioavailability is greater than that before menopause because endogenous estrogen production after menopause occurs primarily in adipose tissue [14, 31]. On the basis of these plausible biological evidence, several previous studies test the interaction between cholesterol consumption and menopausal status, hormone replacement therapy, and body mass index [14, 15, 28]. Although no statistical interaction was observed, Biel et al. found a stronger increased risk with dietary cholesterol among postmenopausal women, particularly those not exposed to hormone replacement therapy, and among overweight and obese women, which was consistent with this hypothesis [14]. Lucenteforte et al. found a significant interaction between cholesterol and hormone replacement therapy, and the association with cholesterol appeared to be confined to nonusers of hormone replacement therapy [15]. However, Goodman et al. did not observe a statistical significant interaction between cholesterol and body mass index [28]. More studies are needed to clarify these interactions in the future.

Despite the publication of a meta-analysis investigating the association between dietary lipids and EC risk in 2007 [24], we decided to conduct this new meta-analysis considering the following points. The previous meta-analysis reported the summary OR was 1.39 (95% CI = 0.97–2.00) which was based on the highest comparing with the lowest category of intake. Since the definitions of the categories differed among these studies, this finding was hard to interpret. For example, McCann et al. [21] reported the highest category (the fourth quartile) of cholesterol consumption was over 427 mg/day in a population-based case-control study with 232 cases and 639 controls in 2000. By comparison, the amount of cholesterol consumption for the fourth quartile was just over 245 mg/day with same study design and country in 1993 [16]. Additionally, they provided the summary estimates for per 150 mg/1000 kcal of cholesterol intake (summary OR = 1.35, 95% CI = 0.96–1.90) which was based on the nutrient density model. Since cholesterol consumption account for a small part of total fat intake, adjustment for total energy intake in the standard multivariate model might have enough power. The previous meta-analysis focused not only on dietary cholesterol but also on other lipids intake. Therefore, the authors only reported summarized risk estimates of these outcomes instead of conducting subgroup analyses to find the source of heterogeneity. Additionally, it is not clear whether the findings of the study were robust in the subgroup and sensitivity analyses. (iii) We have also included five new studies (one cohort and four case-control studies) that greatly increased the statistical power to indentify the association between cholesterol consumption and EC risk. These studies accounted for 55.95% of the weight when evaluating the aforementioned association.

We acknowledge several limitations of our meta-analyses. Compared with randomized controlled trials, observational studies are more likely to inherent susceptible bias. High cholesterol consumption in diets may be related to higher body mass index, cigarette smoking and alcohol drinking, physical activity, and intake of total energy and other nutrients [13, 32]. However, controlling for these potential confounders in the statistical analysis varied between these included studies, raising the possibility that the association between dietary cholesterol consumption and risk of EC was partially the result of unmeasured confounding. The direction of these results of subgroup analyses stratified by adjustment for potential confounders were persisted, though part of them showed statistical significance. In addition, although the results of meta-regression analyses indicated that whether adjustment for these potential confounders might not be the source of heterogeneity, high heterogeneity was still observed in several subgroups which might be attributed to the limited numbers of included studies. Second, the differences between studies design of these included studies also limited us to interpret the findings of this meta-analysis. Only Merritt et al. [17] provided the evidence of cholesterol consumption and EC risk on the basis of the European Prospective Investigation into Cancer and Nutrition (EPIC) study. Case-control studies are inherently more susceptible to bias (e.g., selection bias and recall bias) than cohort studies [33, 34]. Therefore, further prospective cohort studies are warranted to validate the findings of this meta-analysis. Thirdly, food frequency questionnaire (FFQ) was used to assessed the dietary intake in all included studies. However, the number of items of FFQ were varied among these studies, which might generate the heterogeneity. For example, a hospital-based case-control study in 2009 measured the dietary information only based on a FFQ with 44-items [18]. In contrast, the FFQ of the EPIC contained up to 260 food items [17]. Additionally, measurement error on the FFQ is a concern common to observational studies of diet. However, none of these studies corrected the measurement errors which were known to bias effect estimates [35]. The relationship between cholesterol consumption and EC risk could be underestimates by any measurement errors. Last, although there is no evidence of publication bias in the present meta-analysis, tests for publication bias have low statistical power, especially when the number of studies is limited.

In conclusion, results from this meta-analysis indicated that dietary cholesterol consumption was associated with EC among case-control studies, but was not associated with risk in cohort studies. Large prospective studies with better adjustment for potential confounders and are warranted to confirm the aforementioned association. Additionally, further evaluation of the impact of measurement errors on these risk estimates is also warranted.

## MATERIALS AND METHODS

### Databases, sources, and searches

Two investigators (T-TG and Q-JW) systematically and independently searched PubMed, EMBASE, and Web of Science from the database inception to the end of June 2015 for epidemiological studies without restriction. The following search keywords were used: (diet OR dietary OR fat OR cholesterol) AND (endometrium OR endometrial) AND (cancer OR tumor OR carcinoma OR neoplasm). This search strategy was validated in our previous meta-analyses [13, 36–40]. We also hand searched the bibliographies of all the included studies and to identify any remaining studies. We followed the Preferred reporting items for systematic reviews and meta-analyses (PRISMA) guidelines to plan, conduct and report this meta-analysis [41] (Supplementary Table S1).

### Study selection and exclusion

Original studies were included if they (i) had an observational study design; (ii) evaluated the association between cholesterol consumption and EC risk; and (iii) presented relative risk (RR) or OR estimates with 95% CIs or necessary data for calculation [36]; Original studies were excluded if they (i) were randomized controlled trials, reviews without original data, ecological studies, editorials, and case reports; (ii) reported the risk estimates that could not be summarized (such as reported the risk estimates without 95% CIs); and (iii) reported the outcome as EC mortality or recurrence [36]. If several publications involved overlapped individuals, we included the study with the most patients.

### Data extraction and quality assessment

Two investigators (T-TG and Q-JW) extracted the data of these included studies. A third reviewer (Y-ZW) was involved to resolve all the differences. From each eligible study, these two investigators extracted information independently on first author, year of publication, geographic location, the number of cases and controls (in case-control studies) or the number of cases and population participants (in cohort studies), exposure assessment and categories, and study-specific adjusted estimates with their 95% CIs for the highest compared with the lowest category of intake (including adjusted confounders information if applicable). If there were multiple estimates for the association, we used the estimate adjusted for the most appropriate confounding variables, like previous studies [13, 33, 36–38, 42, 43]. The Newcastle-Ottawa Scale (NOS) [33, 34, 36, 44, 45] was used to assess the methodological quality of all included studies.

### Statistical analysis

Since the relative rarity of EC in the general population as well as only one cohort study [17] was included in this analysis, therefore, we interpreted all risk estimates as OR for simplicity [36]. For study [22] did not provide the adjusted risk estimate, we used the exposure distribution of cases and controls to calculate the crude risk estimate.

To examine the associations between the cholesterol consumption and EC risk, the summary OR with 95% CIs were estimated by summarizing the risk estimates of each study using the random effect models (Stata META command) because the summaries of random effect model are relatively more conservative than fixed effect models [46]. We summarized the study-specific OR for each 100 mg/day increment in cholesterol consumption which was recommend by the WCRF/AICR. The study-specific trend from the correlated log RR across the categories of cholesterol consumption was computed by using the generalized least-squares trend estimation method developed by Greenland and Longnecker [47] and Orsini et al. [48] (Stata GLST command). For studies reported the risk estimates as per standard deviation (SD) increment of cholesterol consumption, we used previously described methods [49, 50] to recalculate risk estimates into per 100 mg/day increment. Furthermore, a potential nonlinear dose-response relationship between the cholesterol consumption and the EC risk was modeled by using restricted cubic splines with three knots at fixed percentiles (10, 50 and 90%) of the distribution of exposure [36, 42, 51, 52]. We calculated the overall *P*-value by testing that these two regression coefficients were simultaneously equal to zero. We calculated a *P*-value for nonlinearity by testing that the coefficient of the second spline was equal to zero. The details of this method has been published elsewhere [53, 54].

For conducting the dose-response meta-analysis, the following information were needed: (i) the distribution of cases and non-cases and the risk estimates with the variance estimates for at least three quantitative exposure categories; (2) the median or mean level of these exposures in each category (if reported by ranges, mean level was calculated by averaging the lower and upper bound; if the lowest category was open ended, the lowest boundary was considered to be zero; if the highest category was open ended, the open-ended interval length was assumed to be the same as the adjacent interval). Given this, ten studies met the criteria and were included in the dose-response analysis of cholesterol consumption and EC risk.

To investigate the possible sources of heterogeneity of main results, we carried out stratified analyses by the following study features: study design (cohort *versus* case-control studies), type of control subjects (population-based *versus* hospital-based), geographic location (North America *versus* Europe), validated food frequency questionnaire (yes *versus* no), number of EOC cases which was categorized by the mean value (≥ 450 versus < 450), and adjustment for potential confounders including total energy intake, body mass index, cigarette smoking, parity, oral contraceptive use, menopausal status, and hormone replacement therapy use. Heterogeneity between subgroups was evaluated by meta-regression (Stata METAREG command) [33, 34, 36, 44].

Small study bias, such as publication bias was evaluated with Egger's regression asymmetry test [55] and Begg's rank-correlation test [56] (Stata METABIAS command). A *P*-value of 0.05 was used to determine whether significant publication bias existed. Additionally, sensitivity analyses were conducted by deleting each study in turn to reflect the influence of individual data on the overall estimate (Stata METAINF command). All statistical analyses were performed with Stata (version 12; StataCorp, College Station, TX).

## SUPPLEMENTARY MATERIALS FIGURE AND TABLE


